# Supplementary material for: PMMA/SWCNT Composites with Very Low Electrical Percolation Threshold by Direct Incorporation and Masterbatch Dilution and Characterization of Electrical and Thermoelectrical Properties
Source: Nanomaterials (Basel). 2023 Apr 21;13(8):1431. doi: 10.3390/nano13081431 (PMC10145481; doi:10.3390/nano13081431)
Supplement: Supplementary file 1 [file nanomaterials-13-01431-s001.zip › nanomaterials-2333510-supplementary.pdf]

Supporting informations

PMMA/SWCNT Composites with Very Low Electrical Percolation Threshold by Direct Incorporation and Masterbatch Dilution and Characterization of Electrical and Thermoelectrical Properties

Ezgi Uçar [1,2], Mustafa Dogu [3], Elcin Demirhan [2] and Beate Krause [1,*]

1 Leibniz-Institut für Polymerforschung Dresden e.V. (IPF), Hohe Str. 6, 01069 Dresden, Germany; ezgiucar@gmail.com
2 Chemical Engineering Department, Yildiz Technical University, Davutpasa Campus, Esenler, 34220 Istanbul, Türkiye; demirhan@yildiz.edu.tr
3 Mir Ar-Ge Inc., Research Department, Esenyurt, 34522 Istanbul, Türkiye; mus.dogu@gmail.com
* Correspondence: krause-beate@ipfdd.de; Tel.: +49-351-4658-736

Table S1. Thermoelectric properties of PMMA/SWCNT composites

SWCNT content wt%	Seebeck coefficient µV/K	Volume conductivity S/m	Power factor µW/(m·K²)
0.25	50.6 ± 4.3	2.3 ± 0.0	0.006
0.5	51.4 ± 4.4	4.3 ± 0.4	0.011
1	51.6 ± 0.7	17.4 ± 2.8	0.046
2	53.4 ± 2.2	57.2 ± 9.6	0.163
3	46.3 ± 4.2	96.7 ± 4.2	0.207
5	35.8 ± 1.0	144.5 ± 38.0	0.185

Table S2. Comparison of glass transition temperature Tg values of composites with the change of SWCNT (Tuball) amount

Sample	Tg (°C)
PMMA -250+direct	113.7
PMMA+0.05 wt%Tuball-250+direct	114.6
PMMA+0.075 wt%Tuball-250+direct	114.5
PMMA+0.1 wt%Tuball-250+direct	115.0
PMMA+0.2 wt%Tuball-250+direct	114.5
PMMA+0.25 wt%Tuball-250+direct	115.8
PMMA+0.5 wt%Tuball-250+direct	115.7
PMMA+1 wt%Tuball-250+direct	115.6
PMMA+2 wt%Tuball-250+direct	116.1
PMMA+3 wt%Tuball-250+direct	116.6
PMMA+5 wt%Tuball-250+direct	117.0

Table S3. Comparison of glass transition temperature Tg values of composites prepared at different rotation speeds

Sample	Tg (°C)
PMMA+0.1 wt%Tuball-250+direct	115.5
PMMA+0.1 wt%Tuball -200+direct	113.9
PMMA+0.1 wt%Tuball-150+direct	114.9
PMMA+0.1 wt%Tuball-100+direct	114.6
PMMA+0.1 wt%Tuball -50+direct	114.4
PMMA+1 wt%Tuball-250+direct	116.4
PMMA+1 wt%Tuball-200+direct	115.7
PMMA+1 wt%Tuball-150+direct	115.2
PMMA+1 wt%Tuball-100+direct	114.8
PMMA+1 wt%Tuball-50+direct	114.6
PMMA+0.25 wt%Tuball-250+direct	116.2
PMMA+0.25 wt%Tuball-200+direct	115.1
PMMA+0.25 wt%Tuball-150+direct	114.0
PMMA+0.25 wt%Tuball-100+direct	113.9
PMMA+0.25 wt%Tuball-50+direct	113.9
